# Optimization of Enzymatic Parameters for Enhancing Branch Density and Flow Properties of Sweet Potato Starch

**DOI:** 10.1002/fsn3.70822

**Published:** 2025-09-01

**Authors:** Yang Jiao, Pengtao Wang, Lei Niu, Rong Ai, Liyun Xin, Aili Song, Guoxing Yang, Kai Zhang

**Affiliations:** ^1^ College of Life Science and Engineering Hexi University Zhangye Gansu Province China; ^2^ Gulang Tianyuan Agricultural Industry co., Ltd. Wuwei Gansu Province China

**Keywords:** branch density, enzymatic modification, response surface methodology, rheological properties, sweet potato starch

## Abstract

Enhancing the branch density of starch through enzymatic modification is critical for improving its functional properties in various industrial applications. This study optimized the sequential enzymatic treatment of sweet potato starch using α‐amylase (AA), β‐amylase (BA), and transglucosidase (TG) to maximize the degree of branching (DB). Response Surface Methodology (RSM) was employed to evaluate the synergistic effects of enzyme concentrations and hydrolysis durations, identifying optimal conditions: AA (20.00 U/g, 9.01 h), BA (3.00 U/g, 5.03 h), and TG (2179.06 U/g, 9.00 h). Under these conditions, the DB reached 53.38%, which was within 2.53% of the predicted value (55.91%). Structural analyses via XRD and FTIR revealed reduced crystallinity, indicating disrupted molecular order. The modified starches exhibited significantly enhanced solubility compared to native starch, particularly those with DB values above 40%, attributed to shorter glucan chains and inhibited amylose recrystallization. Rheological studies demonstrated shear‐thinning behavior and diminished viscoelasticity, suggesting improved flow properties for low‐viscosity applications. These findings highlight the efficacy of enzymatic combinatorial modification in tailoring starch functionality, offering a promising approach for producing high‐branch‐density starches with enhanced flow and solubility properties for use in sauces, beverages, and biodegradable films.

## Graphical abstract

This study optimized the sequential enzymatic treatment of sweet potato starch using α‐amylase, β‐amylase, and transglucosidase to enhance its branch density, achieving a DB of 53.38%. Structural and physicochemical analyses showed reduced crystallinity, improved solubility, and lower viscosity, contributing to better hydrodynamic properties. These results demonstrate the potential of combinatorial enzyme modification to tailor starch functionality for food and industrial applications.

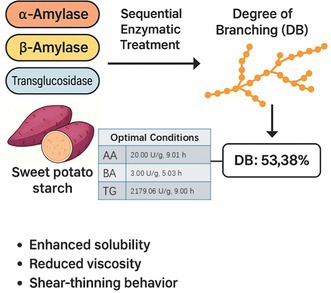

## 1 Introduction

Starch has been widely utilized in various food applications, including soups, sauces, gravies, baked goods, dairy confections, snacks, batters, coatings, and meat products. Beyond the food industry, starch also plays a crucial role in pharmaceuticals, textiles, bioethanol production, adhesives, low‐calorie substitutes, biodegradable packaging materials, thin films, and thermoplastic materials (Tharanathan 2005; Kaur et al. 2012). Starch typically consists of 20%–30% linear amylose and 70%–80% highly branched amylopectin. Amylose is primarily composed of linear chains of α‐(1 → 4)‐linked D‐glucose units, with occasional branching. In contrast, amylopectin exhibits a complex branched structure, comprising α‐(1 → 4)‐linked D‐glucan chains with α‐(1 → 6)‐linked D‐glucose branches that aggregate into clustered formations. The outer chains of these clusters can adopt a double‐helical structure within crystalline lamellae, while the inner chains, located between branch points, contribute to the formation of amorphous lamellae (Bezerra et al. 2008; Jane 2009). Recent studies have increasingly highlighted the pivotal role of amylopectin's branch structure in determining the physicochemical and digestive properties of granular starch. Generally, a higher branch density significantly enhances starch solubility (Sorndech et al. 2015). Additionally, increased branch density and shorter branch chain lengths lead to a reduction in retrogradation enthalpy, indicating a slower retrogradation process (Singh et al. 2012). Amylopectin with a greater branch density and a higher proportion of short chains exhibits lower susceptibility to enzymatic hydrolysis by amylases, which may offer health‐related benefits (Naguleswaran et al. 2014). Furthermore, an increased number of α −1,6 branch points in starch paste have been shown to reduce both peak viscosity and setback values. Notably, gelatinization enthalpy exhibits a linear correlation with the content of α −1,6 branch points (Ren et al. 2017).

The branch structure of starch plays a critical role in its industrial applications, and efforts to enhance its versatility by increasing branch density have been extensively investigated (Tharanathan 2005). Recent studies have focused on modifying the internal chain structure of amylopectin to increase branch density using β‐amylase and transglucosidase (Miao et al. 2014; Shah et al. 2018; Shi et al. 2014). These transferase‐based modifications primarily catalyze the hydrolysis of α −1,4‐linked linear chains in amylose and amylopectin, followed by a transglycosylation reaction to generate new α −1,6‐linked branch chains, thereby increasing branch density (Ao et al. 2007; van der Maarel and Leemhuis 2013). In our preliminary experiments, we found that compared to using β‐amylase (BA) and transglucosidase (TG) alone, the combination of α‐amylase (AA), BA, and TG resulted in modified starch with a higher degree of branching. However, limited research has been conducted on starch modification using this enzymatic combination. Moreover, variations in the concentrations and hydrolysis durations of AA and BA significantly influenced the degree of branching (DB) of starch. Yet, how to optimize the hydrolytic conditions of AA and BA to enhance starch branch density remains largely unexplored. Therefore, this study investigated the efficiency of α −1,6 branch formation using a sequential AA→BA→TG enzymatic treatment.

Response Surface Methodology (RSM), a powerful statistical tool for modeling complex systems, was employed to assess the combined effects of multiple factors and determine the optimal conditions for achieving the desired modifications (Bezerra et al. 2008). The primary objective was to optimize enzyme concentrations and hydrolysis times to produce modified sweet potato starches with varying degrees of branching (DB) and to refine enzymatic conditions using RSM for the production of starch with enhanced DB. Additionally, the hydrodynamic properties of modified sweet potato starches with different DB values were investigated, providing novel insights that may facilitate the development of starches with higher branch densities.

## 2 Materials and Methods

### 2.1 Materials

Sweet potato starch was obtained from Jingyuchao Food Co. (Shandong, China). α‐Amylase from porcine pancreatin (AA, 50 U/mg, Pcode: 101574854) and barley β‐amylase (BA, 53 U/mg, Pcode: 1002058163) were purchased from Sigma‐Aldrich. Promozyme D2 (pullulanase, 1350 U/mL) was generously provided by Novozymes (Denmark). Transglucosidase L from *Aspergillus niger* (TG, 25,000 U/mL) was a kind gift from Amano Enzyme Inc. (Japan). Isoamylase (1000 U/mL) was obtained from Megazyme (USA). Amylase activity was assessed by quantifying reducing sugars released from 1.0% soluble starch using the Somogyi–Nelson method (Somogyi 1952).

### 2.2 Preparation of Triple Enzyme‐Modified Starch

#### 2.2.1 Experimental Design of Single Factor

A sequential enzymatic modification approach (AA → BA → TG) was chosen based on preliminary results, which demonstrated superior branching efficiency compared to individual or simultaneous enzyme treatments. The rationale lies in the potential synergistic action: α‐amylase primarily cleaves internal α‐(1,4) bonds, facilitating better substrate access for β‐amylase, which releases maltose units. Subsequently, transglucosidase utilizes these shorter chains to introduce α‐(1,6) branches, effectively enhancing the overall branch density.

Sweet potato starch (10 g) was suspended in 200 mL of 0.02 M sodium acetate buffer (pH 6.9) containing 0.2 mL of 10% sodium azide solution. The suspension was heated in a boiling water bath with continuous magnetic stirring (350 rpm) for 30 min to ensure complete gelatinization, followed by cooling to 50°C. The gelatinized starch dispersions (pH 6.9, optimal pH) were then incubated with AA at concentrations of 2, 4, 8, 16, 24, and 32 U/g dry starch at 50°C (optimal temperature) under continuous stirring for different durations (2, 4, 6, 8, 10, and 12 h). After incubation, the reaction was terminated by heating the samples in a boiling water bath for 30 min.

Next, the pH of the AA‐treated starch dispersions was adjusted to 5.2 (optimal pH) using 0.02 M acetic acid buffer solution. These dispersions were then incubated with BA at concentrations of 2, 4, 8, 16, 24, and 32 U/g dry starch at 37°C (optimal temperature) under continuous stirring for various durations (2, 4, 6, 8, 10, and 12 h). Following incubation, the enzymatic reaction was terminated by heating in a boiling water bath for 30 min.

Subsequently, the pH of the AABA‐treated starch dispersions was adjusted to 5.0 (optimal pH) before incubation with TG at concentrations of 1500, 3000, 4500, 6000, 7500, and 9000 U/g dry starch at 55°C under continuous stirring for different time intervals (2, 4, 6, 8, 10, and 12 h). The reaction was then stopped by heating the samples in a boiling water bath for 30 min.

The resulting starch dispersions were subjected to dialysis (MWCO: 3500) in deionized water to remove released oligosaccharides (primarily maltose) and residual salts from the buffer solution. To precipitate the modified starch, three volumes of 90% ethanol (v/v) were added. The precipitated samples were collected by centrifugation at 5000 g for 10 min, washed with deionized water, freeze‐dried, ground, and sieved through a 100‐mesh screen. The modified starch samples were then stored in sealed bags for DB analysis. The optimal levels of each factor were determined based on the DB analysis.

#### 2.2.2 Response Surface Methodology

In this study, response surface methodology (RSM) was employed to design experiments aimed at evaluating the effects of six key parameters on the DB of starch. Based on preliminary single‐factor experiments, a Box–Behnken design (BBD) was utilized to optimize the experimental conditions. The investigated parameters included α‐amylase concentration (X1), β‐amylase concentration (X2), transglucosidase concentration (X3), α‐amylase hydrolysis time (X4), β‐amylase hydrolysis time (X5), and transglucosidase hydrolysis time (X6), as shown in Table 1.

**TABLE 1:** Variables and experimental design levels for response surface.

Independent variables	Coded symbols	Levels		
		−1	0	1
α‐amylase concentration/(u/g)	X1	12	16	20
β‐amylase concentration/(u/g)	X2	1	2	3
Transglucosidase concentration/(u/g)	X3	500	1500	2500
α‐amylase hydrolytic time/h	X4	9	10	11
β‐amylase hydrolytic time/h	X5	5	6	7
Transglucosidase hydrolytic time/h	X6	9	10	11

The selection of enzyme concentrations and hydrolysis times for the RSM design was based on the results of single‐factor experiments. These preliminary tests revealed the approximate range within which each enzyme exerted the most significant effect on DB. The chosen levels ensured that the response surface could adequately capture both the main effects and interactions of each parameter on starch branching.

#### 2.2.3 Determination of Degree of Branching (DB)

DB analysis was performed following previously established methods (Biliaderis et al. 1981). The change in reducing sugar content was determined using the 3,5‐dinitrosalicylic acid (DNS) method. The reducing power of starch debranched by isoamylase (Megazyme, Bray, Ireland) was analyzed.

For the analysis, a 10 mg sample was dissolved in a mixture of 100 μL of 1 M sodium hydroxide and 200 μL of distilled water, followed by boiling for 30 min. The pH was then adjusted to 7.0 using 1 M hydrochloric acid, and the solution was diluted with distilled water to a final volume of 1 mL. A 0.5 mL aliquot of the sample solution was transferred to a microtube, and 0.50 mL of 50 mM sodium acetate buffer (pH 4.3) along with 10 μL of isoamylase was added. The mixture was incubated at 45°C for 2 h, and the reaction was subsequently terminated by boiling for 10 min. Following this, 0.5 mL of the reaction mixture was transferred to a microtube, and 0.5 mL of DNS reagent was added. The solution was boiled for 5 min and then rapidly cooled. Absorbance was measured at 575 nm.

### 2.3 Fourier Transforms Infrared (FTIR) Spectroscopy

The FTIR spectra of native and modified sweet potato starches were obtained using an FTIR spectrophotometer (Perkin Elmer, SA) within the range of 500–4000 cm^−1^ at a resolution of 4 cm^−1^. Spectra were baseline corrected. The ground samples were mixed with dried KBr (1:100 w/w) and then pellets were prepared by compression and analyzed.

### 2.4 X‐Ray Diffraction (XRD)

The crystallinity of native and modified starches was analyzed using a Bruker D8 Discover A25 X‐ray diffractometer (Bruker AXS, Rheinfelden, Germany) equipped with a copper tube operating at 40 kV and 40 mA, emitting Cu Kα radiation with a wavelength of 0.154 nm. A divergence slit of 1° and a receiving slit of 0.3 mm were used. The diffraction patterns were recorded in the 2θ range of 5° to 40° with a scanning rate of 2°min and a step size of 0.05°. The moisture content of the powdered starch samples for XRD analysis was adjusted to (25.00% ± 0.03%) by storing them in a desiccator over saturated K₂SO₄ at 25°C for 10 days. No further increase in moisture content was observed upon extended storage. The relative crystallinity (RC, %) of each starch sample was determined as the ratio of the crystalline peak area to the total diffractogram area, calculated using Jade 5.0 software (Materials Data Inc., Livermore, CA, USA) following the method of Hermans and Weidinger (Hermans and Weidinger 1948).

### 2.5 Solubility Analysis

The solubility (%) of native and modified starches was determined following a previously described method with slight modifications (Kim et al. 2015). Briefly, a starch sample (0.5 g, dry basis) was mixed with 25 mL of double‐distilled water in a 50 mL centrifuge tube and vortexed at 25°C for 5 min. The mixture was then centrifuged at 3000 × g for 20 min. The supernatant was carefully collected, while the residue was weighed. The collected supernatant was dried in an air‐drying oven at 105°C until a constant weight was achieved, and then weighed. The solubility (%) of native and modified starches was calculated using the following equations:

$$\begin{array}{c} \text{Solubility}\;\left(\%\right)\\ =\left(\text{dried supernatant weight}/\text{weight of}\;\mathrm{dry}\;\text{starch}\right)\times 100 \end{array}$$

### 2.6 Rheological Properties

#### 2.6.1 Steady Shear Rheological Analysis

Dispersions of native and modified starch (6.0% w/v) were heated in a boiling water bath with continuous magnetic stirring for 30 min, then rapidly cooled to 25°C for immediate measurement (a starch concentration of 6.0% (w/v) was selected based on prior studies demonstrating that this level provides a balance between measurement sensitivity and practical relevance for food applications). The steady shear rheological properties were analyzed using a DHR‐1 rotational rheometer (TA Instruments, New Castle, USA) equipped with a 40‐mm cone‐plate geometry, a gap of 0.05 mm, and a cone angle of 2° at 25°C. The apparent viscosity‐shear rate behavior was determined by increasing the shear rate from 0 to 300 s^−1^ at 25°C. The starch dispersion was loaded onto the rheometer platen, and the exposed sample edges were covered with a thin layer of light paraffin oil to minimize evaporation during measurements.

#### 2.6.2 Dynamic Viscoelasticity Analysis

Dynamic viscoelasticity was assessed following previously established methods with minor modifications (Min et al. 2023). Native and modified starch dispersions (6.0% w/v) were heated in a boiling water bath with continuous magnetic stirring for 30 min, then rapidly cooled to 25°C for immediate measurement. Dynamic shear properties were analyzed using a DHR‐1 rotational rheometer (TA Instruments, New Castle, USA) equipped with a 40‐mm cone‐plate geometry and a Peltier temperature controller. The sample was placed on the rheometer platen, and a thin layer of light paraffin oil was applied to the exposed sample edges to prevent evaporation during measurement. The dependence of complex dynamic viscosity (η*), storage modulus (G′), loss modulus (G″), and loss tangent (Tan δ = G″/G′) on angular frequency (ω) was determined over a frequency range of 0.1 to 100 rad/s, with a strain amplitude set within the linear viscoelastic region at 30%.

### 2.7 Statistical Analysis

To quantify differences between individual treatments over time, statistical analysis was performed using the Statistical Analysis System (SAS) software (version 9.3; SAS Institute, USA). Results were expressed as the mean ± standard deviation (SD) based on triplicate analyses for each sample. Mean comparisons were conducted using the least significant difference (LSD) test. A one‐way analysis of variance (ANOVA) was carried out using ORIGIN 7.5 (OriginLab Inc., USA), followed by Tukey's test to determine significant differences among mean values at a confidence level of 0.05.

Response surface methodology (RSM) was employed to design experiments aimed at investigating the effects of six key parameters on the degree of branching (DB) of starch. Based on single‐factor experiments, a Box–Behnken response surface design (BBD) was utilized to optimize experimental conditions, including enzyme concentrations and hydrolysis time, using the “Design Expert” software (Version 8.0, Stat‐Ease Inc., Minneapolis, USA). RSM was also applied to predict results through isoresponse contour plots and three‐dimensional surface plots. The quality of model fit was assessed using the coefficient of determination (R^2^), while statistical significance was evaluated using the F‐test within the same software.

## 3 Results and Discussion

### 3.1 Statistical Optimization of DB Value Using RSM

#### 3.1.1 Results of Single‐Factor Experiments

**FIGURE 1:**
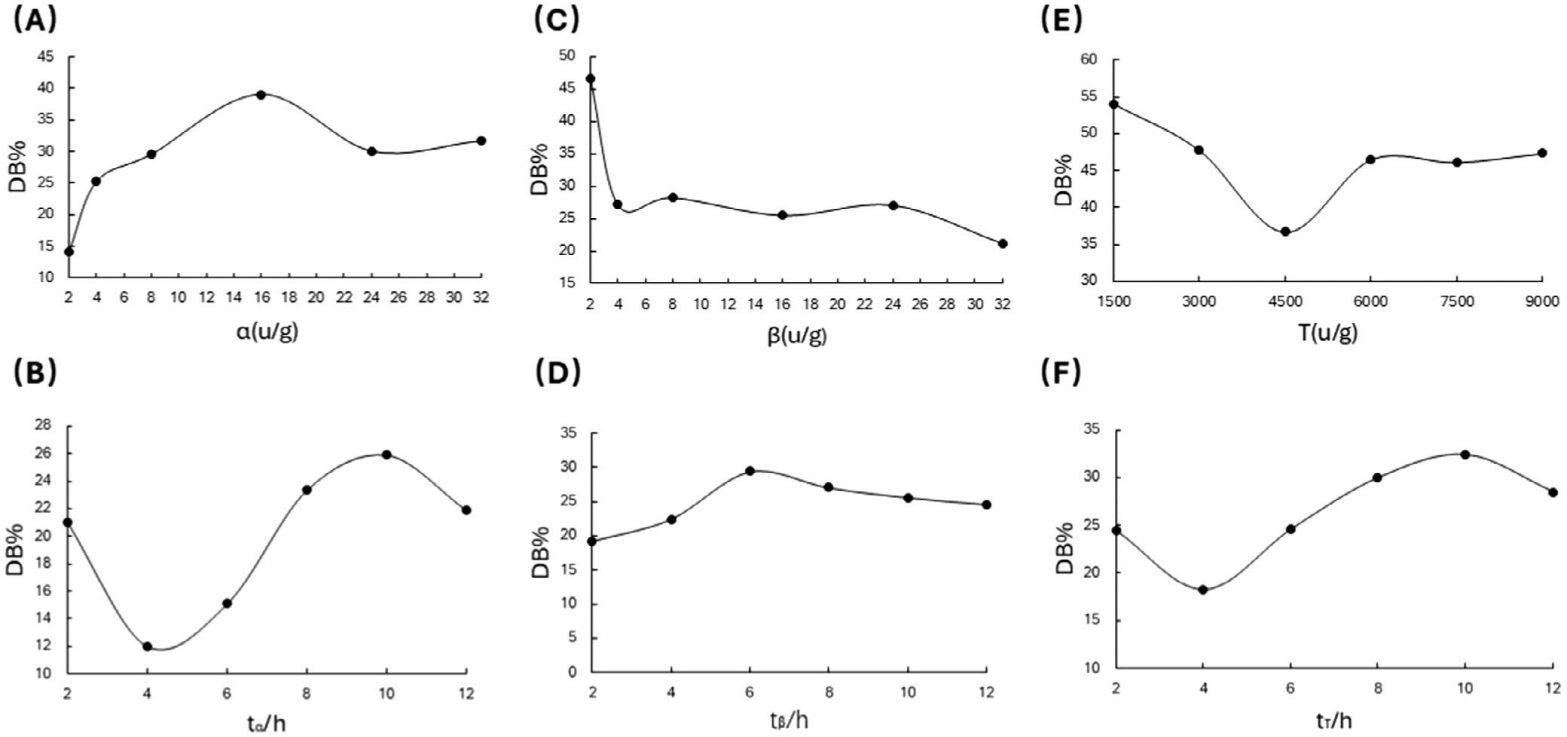
Results of single‐factor experiments. (A, B) Effect of α‐amylase on starch branching degree at different addition amounts and treatment times. (C, D) Effect of β‐amylase on starch branching degree at different addition amounts and treatment times. (E, F) Effect of transglucosidase on starch branching degree at different addition amounts and treatment times.

As shown in Figure 1A,B the degree of branching (DB) of sweet potato starch initially decreased, then increased, and subsequently declined again with the extension of α‐amylase hydrolysis time. The highest DB was observed at a hydrolysis time of 10 h. Similarly, as the α‐amylase dosage increased, the DB first increased and then decreased, reaching its maximum at an enzyme concentration of 16 U/g. Figure 1C,D illustrates that with the prolongation of β‐amylase hydrolysis time, the DB of sweet potato starch first increased and then decreased, peaking at 6 h. As the β‐amylase dosage increased, the DB initially decreased and then stabilized, with the highest DB observed at an enzyme concentration of 2 U/g. As depicted in Figure 1E,F the DB of sweet potato starch first decreased, then increased, and subsequently declined again with the extension of glucosyltransferase hydrolysis time, reaching its maximum at 10 h. As the glucosyltransferase dosage increased, the DB initially decreased, then increased, and gradually stabilized, with the highest DB observed at an enzyme concentration of 1500 U/g.

#### 3.1.2 Mathematical Equations Predicted by the Model

Response surface methodology (RSM) was employed to analyze the correlation between the designed variables and the experimental response. The Box–Behnken design (BBD) of RSM was used to determine the optimal levels of six key factors and to assess their interactive effects on the degree of branching (DB) of starch.

The experimental design matrix (Table 2) includes the key factors: α‐amylase concentration (X1, U/g), β‐amylase concentration (X2, U/g), transglucosidase concentration (X3, U/g), α‐amylase hydrolysis time (X4, h), β‐amylase hydrolysis time (X5, h), and transglucosidase hydrolysis time (X6, h), along with the corresponding DB results. The predicted DB values were obtained by fitting a quadratic model using Design Expert software (Version 8.0). The statistical model was developed based on the experimental DB data through multiple regression analysis and is expressed as follows:

$$\begin{array}{cc} Y\left(\mathrm{DB},\%\right)= & 36.83+1.78X1-0.48X2-0.92X3\\ & +0.06\text{1X}4-0.94X5-0.57X6-2.08X1X2\\ & +0.07X1X3-1.62X1X4-0.82X1X5-0.21X1X6\\ & +0.54X2X3-0.50X2X4+0.34X2X5-1.92X2X6\\ & -0.55X3X4-2.36X3X5-0.21X3X6+3.92X4X5\\ & +2.36X4X6+2.34X5X6-0.69{\text{X1}}^{2}+3.39{\text{X2}}^{2}-0.59{\text{X3}}^{2}\\ & -0.63{\text{X4}}^{2-}0.34{\text{X5}}^{2}+2.00{\text{X6}}^{2} \end{array}$$

**TABLE 2:** Experimental design and response of Box–Behnken.

Run	X1	X2	X3	X4	X5	X6	DB (%)
1	0	0	0	0	0	0	37.60
2	−1	0	1	0	0	1	34.23
3	0	0	0	0	0	0	35.74
4	1	1	0	1	0	0	34.98
5	0	0	−1	−1	0	1	35.00
6	0	0	0	0	0	0	36.06
7	0	0	1	1	0	1	37.99
8	0	1	−1	0	1	0	39.86
9	0	−1	0	0	−1	1	41.74
10	0	0	−1	1	0	−1	38.72
11	0	−1	−1	0	−1	0	40.94
12	1	−1	0	1	0	0	41.51
13	0	0	−1	−1	0	−1	41.21
14	1	1	0	−1	0	0	42.85
15	1	0	−1	0	0	−1	39.92
16	0	1	0	0	−1	0	37.60
17	1	0	−1	0	0	1	40.84
18	0	0	1	1	0	−1	34.03
19	−1	0	−1	0	0	1	37.87
20	0	1	0	0	1	−1	41.87
21	1	0	1	0	0	1	37.69
22	0	1	1	0	−1	0	41.54
23	0	−1	−1	0	1	0	42.17
24	−1	0	−1	0	0	−1	35.89
25	−1	0	0	−1	−1	0	35.79
26	−1	1	0	−1	0	0	37.21
27	1	0	1	0	0	−1	38.98
28	−1	0	0	1	1	0	38.98
29	1	0	0	−1	1	0	32.30
30	0	−1	0	0	−1	−1	45.77
31	1	−1	0	−1	0	0	43.19
32	0	1	0	0	1	1	39.49
33	1	0	0	−1	−1	0	43.08
34	0	0	0	0	0	0	36.86
35	0	1	0	0	−1	−1	45.91
36	0	−1	0	0	1	−1	36.94
37	1	0	0	1	−1	0	33.97
38	0	1	−1	0	−1	0	36.46
39	0	1	1	0	1	0	34.65
40	−1	0	1	0	0	−1	34.88
41	−1	1	0	1	0	0	42.01
42	0	0	0	0	0	0	38.00
43	−1	−1	0	1	0	0	36.03
44	0	0	1	−1	0	−1	40.76
45	−1	0	0	1	−1	0	30.78
46	−1	−1	0	−1	0	0	33.40
47	1	0	0	1	1	0	38.49
48	−1	0	0	−1	1	0	27.93
49	0	−1	0	0	1	1	45.68
50	0	0	−1	1	0	1	39.91
51	0	−1	1	0	1	0	35.65
52	0	0	1	−1	0	1	33.22
53	0	−1	1	0	−1	0	43.00
54	0	0	0	0	0	0	36.69

In the quadratic regression equation, linear terms such as X₁ (α‐amylase concentration) and X₄ (α‐amylase hydrolysis time) showed positive contributions to DB, indicating that higher levels of these variables tended to increase branching. In contrast, terms like X₂ (β‐amylase concentration) and X₅ (β‐amylase hydrolysis time) showed negative coefficients, suggesting their overuse may reduce DB. Interaction terms (e.g., X₁X₂, X₄X₆) and quadratic terms (e.g., X₂^2^, X₆^2^) capture the synergistic and diminishing returns effects, respectively, allowing more precise optimization within the design space.

#### 3.1.3 ANOVA Analysis

Analysis of variance (ANOVA) is essential for evaluating the adequacy and significance of the quadratic model. Generally, the *P*‐value and *F*‐value serve as key indicators for assessing the interaction patterns among variables. A higher *F*‐value and a lower *P*‐value signify greater statistical significance of the corresponding factors. Typically, a factor is considered statistically significant when its *P*‐value is less than 0.05. The ANOVA results are summarized in Table 3.

**TABLE 3:** ANOVA for the response surface model applied.

Source	Sum of squares	Degree of freedom	Mean square	*F*‐value	*P*‐value
Model	721.47	27	26.72	14.71	< 0.0001*
X1‐α	76.33	1	76.33	42.02	< 0.0001*
X2‐β	5.60	1	5.60	3.08	0.0910
X3‐T	20.48	1	20.48	11.27	0.0024*
X4‐t_α_	0.089	1	0.089	0.049	0.8267
X5‐t_β_	21.23	1	21.23	11.68	0.0021*
X6‐t_T_	7.73	1	7.73	4.25	0.0493*
X1 X2	34.69	1	34.69	19.10	0.0002*
X1 X3	0.039	1	0.039	0.022	0.8843
X1 X4	42.06	1	42.06	23.15	< 0.0001*
X1 X5	5.44	1	5.44	3.00	0.0952
X1 X6	0.36	1	0.36	0.20	0.6593
X2 X3	2.34	1	2.34	1.29	0.2664
X2 X4	2.02	1	2.02	1.11	0.3013
X2 X5	1.80	1	1.80	0.99	0.3284
X2 X6	29.64	1	29.64	16.32	0.0083*
X3 X4	2.40	1	2.40	1.32	0.2610
X3 X5	44.51	1	44.51	24.50	< 0.0001*
X3 X6	0.72	1	0.72	0.40	0.5338
X4 X5	122.93	1	122.93	67.67	< 0.0001*
X4 X6	44.65	1	44.65	24.58	< 0.0001*
X5 X6	43.71	1	43.71	24.06	< 0.0001*
X1^2^	4.95	1	4.95	2.72	0.1109
X2^2^	118.34	1	118.34	65.15	< 0.0001*
X3^2^	3.61	1	3.61	1.99	0.1703
X4^2^	4.03	1	4.03	2.22	0.1484
X5^2^	1.19	1	1.19	0.66	0.4251
X6^2^	41.09	1	41.09	22.62	< 0.0001*
Residual	47.23	26	1.82		
Lack of Fit	43.47	21	2.07	2.75	0.1324
Pure Error	3.76	5	0.75		
Cor Total	768.70	53			
R‐Squared	0.9386				
Adj R‐Squared	0.9748				
Pred R‐Squared	0.9575				
Adeq Precision	19.75				
C.V.%	3.53				

* Significant at *p* < 0.05.

The model's *F*‐value of 14.71 indicates that the model is statistically significant, with only a 0.01% probability that such a large *F*‐value could arise due to random noise, thereby confirming the robustness of the model. A *P*‐value of less than 0.05 suggests that the model terms are significant. In this case, X1, X3, X5, X6, X1X2, X1X4, X2X6, X3X5, X4X5, X4X6, X5X6, X2^2^, and X6^2^ were identified as significant model terms.

The lack‐of‐fit *P*‐value was 0.1324, which is greater than 0.05, indicating that the lack of fit was not significant. This suggests that the model provides a good fit for the experimental data. Therefore, the regression equation can be used to replace the actual experimental points for analysis and prediction of the experimental results. The low coefficient of variation (C.V. = 3.53%) and the high coefficient of determination (R^2^ = 0.9386) and adjusted R^2^ (Adj‐R^2^ = 0.9748) confirm that the regression model accurately represents the experimental data with a confidence level exceeding 90%. Furthermore, the “Pred R‐Squared” value of 0.9575 was in reasonable agreement with the “Adj R‐Squared” value of 0.9748. Additionally, the “Adeq Precision” value of 19.75, which measures the signal‐to‐noise ratio, exceeds the threshold of 4, indicating a strong and reliable signal. These results confirm that the model is suitable for navigating the design space.

#### 3.1.4 Normal Plots of Residuals

**FIGURE 2:**
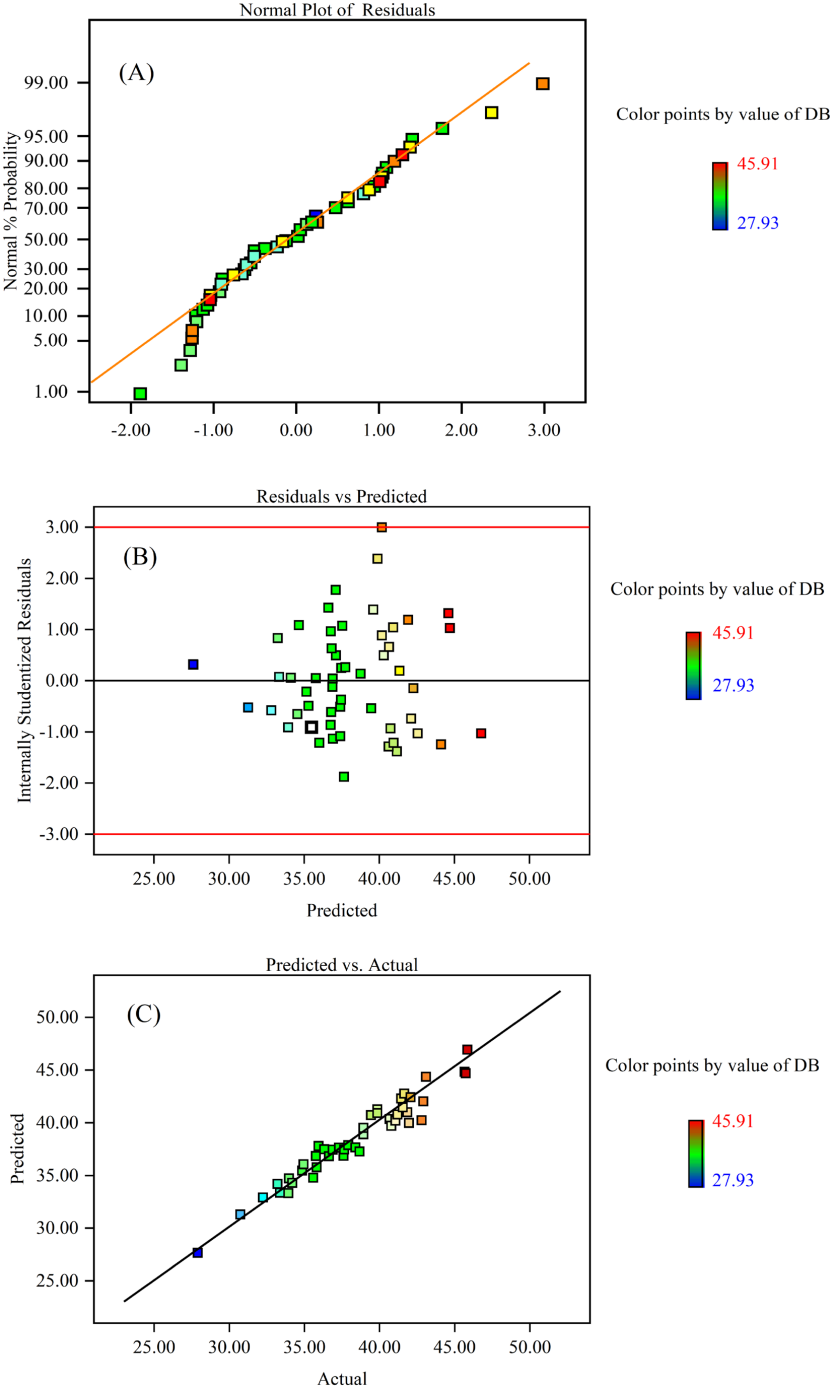
Evaluation of RSM correlation for DB of the modified starch (%). (A) Normal probability plot of the residuals, (B) plot of the residual versus predicted response, (C) plot of the predicted versus actual values.

Residual analysis is an essential method for assessing the validity of an experimental design. The normal probability plot of residuals is shown in Figure 2A, where the data points align approximately along a straight line, indicating that the residuals follow a normal distribution and are independent. Figure 2B illustrates the residuals plotted against the predicted response values, showing a random distribution of points. The residuals form a horizontal pattern, with an equal number of points above and below the horizontal line. Furthermore, all points fall within the ±3.00 range, demonstrating the accuracy and reliability of the proposed model. Figure 2C presents a strong correlation between the experimental data and the model‐predicted values, as the points are closely aligned along the diagonal. This further confirms that the experimental results are in good agreement with the predicted values.

#### 3.1.5 Response Surface and Contour Plots

**FIGURE 3:**
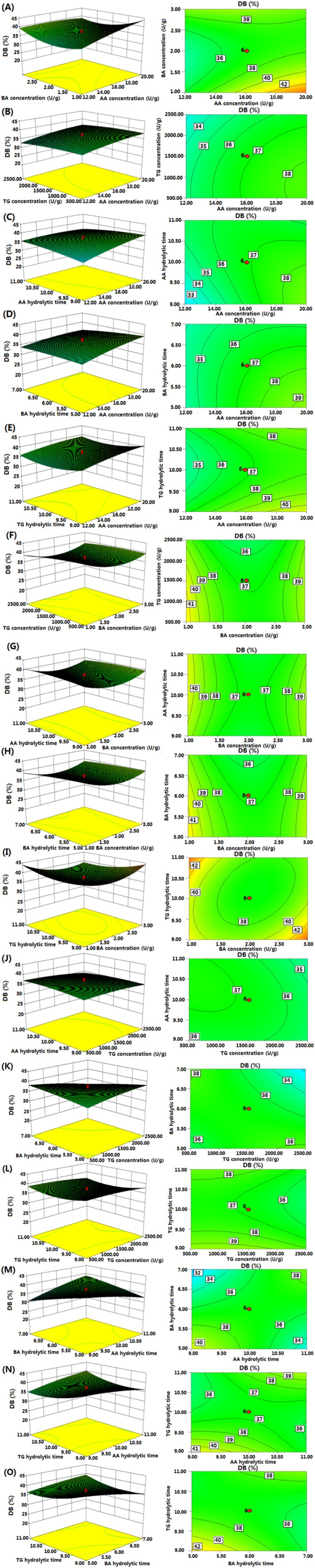
The response surface plots (left) and the corresponding contour lines (right) showing the effects of enzyme concentration and hydrolytic time on degree of branching of sweet potato starch at optimal pH and temperature.

To better visualize the statistically significant factors derived from the above statistical model, three‐dimensional response surface plots illustrating the effects of independent variables on DB values are presented in Figure 3A–O.

Figure 3A shows the interaction between the concentrations of α‐amylase and β‐amylase on DB. As the α‐amylase concentration increases from 12.00% to 20.00%, DB also increases. In contrast, DB decreases with increasing β‐amylase concentration from 1.00% to 1.80%, before slightly rising beyond 1.80%. Figure 3B depicts the interaction between α‐amylase and transglucosidase concentrations, showing that while DB increases with higher α‐amylase concentrations, it decreases with increasing transglucosidase concentrations.

Figure 3C illustrates the significant effects of hydrolysis time and α‐amylase concentration on DB. DB generally increases with both factors, and higher α‐amylase concentrations contribute significantly to an increase in DB. In Figure 3D,E an initial increase in the hydrolysis time of β‐amylase and transglucosidase results in a decrease in DB, followed by a subsequent rise. Figure 3F further confirms that DB decreases with increasing β‐amylase concentration before rising again, while it consistently decreases with increasing transglucosidase concentration.

Figure 3G–L displays the interaction effects of β‐amylase and transglucosidase concentrations with the hydrolysis time of α‐amylase, β‐amylase, or transglucosidase on DB. As the hydrolysis time of β‐amylase increases, DB decreases. However, with increasing transglucosidase hydrolysis time, DB initially decreases before rising. DB increases and then decreases with rising transglucosidase concentration. Figure 3M–O illustrates the effects of the hydrolysis times of α‐amylase, β‐amylase, and transglucosidase on DB. In each case, DB initially decreases before increasing as hydrolysis time extends.

The optimal parameters for DB were determined using Design‐Expert 8.0 software. The optimal conditions were as follows: α‐amylase concentration of 20.00 μg/g, α‐amylase hydrolysis time of 9.01 h, β‐amylase concentration of 3.00 μg/g, β‐amylase hydrolysis time of 5.03 h, transglucosidase concentration of 2179.06 μg/g, and transglucosidase hydrolysis time of 9.00 h. Under these conditions, the model predicted a DB value of 55.91%. To validate the predictive accuracy of the model, a separate optimization experiment was conducted using the predicted optimal conditions. In contrast, experimental replication refers to performing each designed experiment in triplicate to ensure reproducibility and statistical reliability. These two processes together confirmed both the accuracy and robustness of the model predictions. The experimentally determined DB value under these optimal conditions was 53.38%, closely matching the predicted value.

In summary, the response surface analysis provided clear insights into the main and interactive effects of enzymatic parameters, supporting the selection of optimal treatment conditions to maximize DB with high predictive accuracy.

### 3.2 FTIR Spectroscopy

**FIGURE 4:**
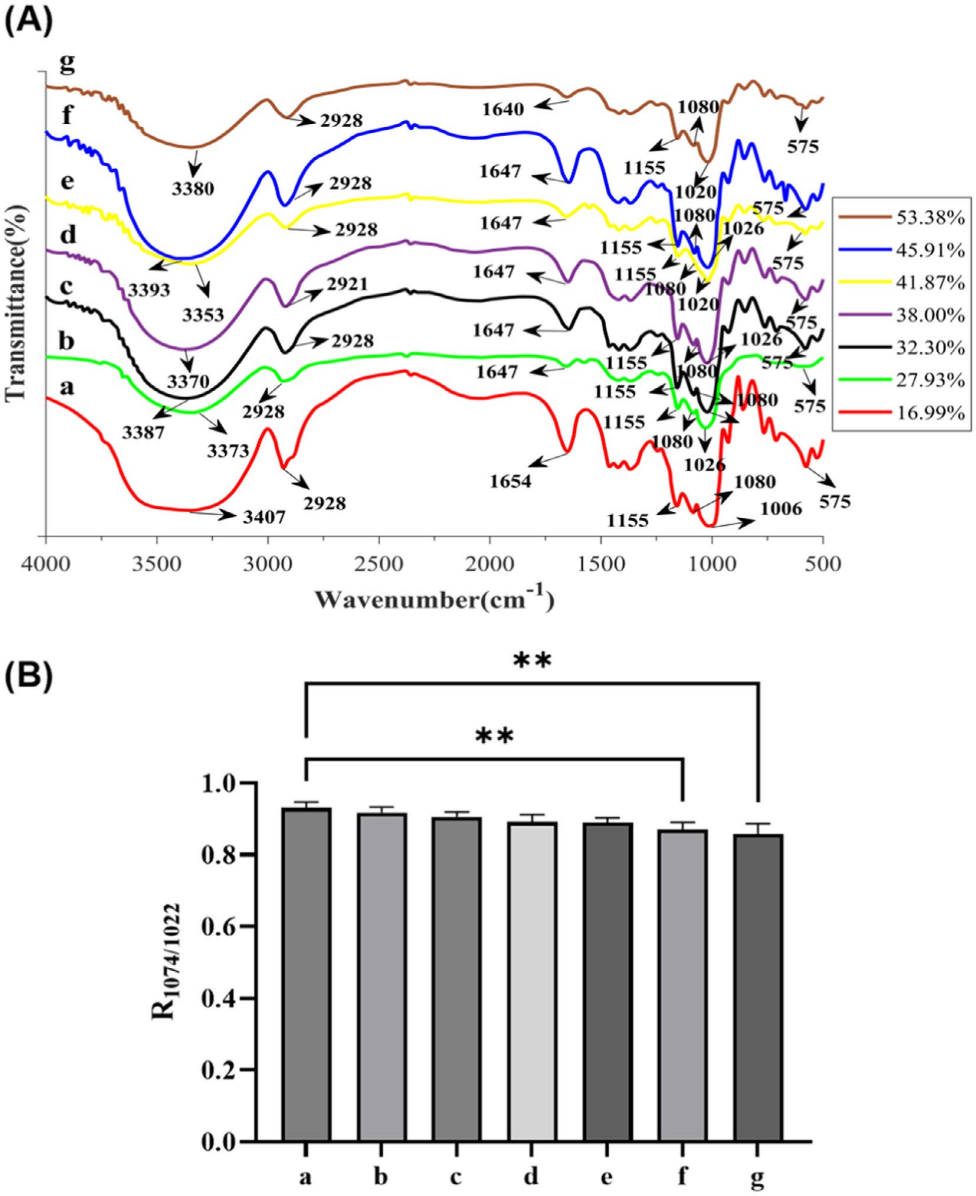
The FTIR spectra (A) and R1047/1022 (B). (a. 16.99% (control), b. 27.93%, c. 32.30%, d. 38.00%, e. 41.87%, f. 45.91%, g. 53.38%).

The FTIR spectra of native and modified sweet potato starches exhibited an absorption peak at 3380 cm^−1^, corresponding to the –OH stretching vibration of glucose units. A strong peak at 2929 cm^−1^ was attributed to the stretching vibrations of CH₂ functional groups. Absorption peaks around 1500 and 1600 cm^−1^ were associated with C–O–C and –COO functional groups (Gani et al. 2016). Enzymatic treatment of the starch led to a shift of absorption peaks at 3400, 2929, 1047, and 995 cm^−1^ toward lower wavenumbers, indicating a reduction in hydrogen bonding strength among starch molecules. Additionally, the bands at 1047 cm^−1^ are indicative of crystalline regions, whereas the absorption peak at 1022 cm^−1^ is characteristic of amorphous domains (Mutungi et al. 2011). Therefore, the intensity ratios of 1047 cm^−1^/1022 cm^−1^ can be used to assess the molecular order of starches. These ratios were found to decrease following enzymatic modification, likely due to the substitution of α‐(1,4)‐glycosidic bonds with α‐(1,6)‐glycosidic bonds, which disrupted molecular packing and increased the proportion of amorphous regions Figure 4A,B. This reduction in molecular order was consistent with the findings from X‐ray diffraction (XRD) analysis.

Overall, the FTIR analysis revealed that enzymatic modification disrupted hydrogen bonding and molecular order in starch, consistent with the observed increase in amorphous regions and branching.

### 3.3 XRD

**FIGURE 5:**
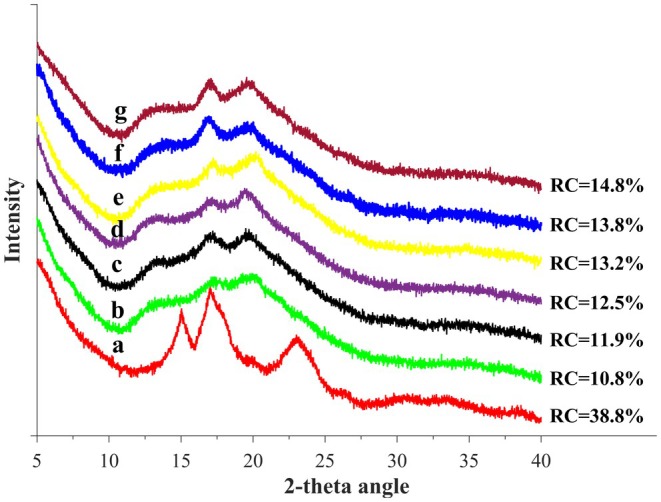
X‐ray diffraction patterns of the native sweet potato starch and enzyme‐treated sweet potato starches: (a) 16.99% (control), (b) 27.93%, (c) 32.30%, (d) 38.00%, (e) 41.87%, (f) 45.91%, (g) 53.38%.

Figure 5 presents the X‐ray diffraction (XRD) patterns of the starch samples. As illustrated in Figure 5, native sweet potato starch exhibits a Ca‐type crystalline structure, characterized by strong diffraction peaks at 2θ values of 15.0°, 17.2°, 18.2°, and 23.5°, along with a weaker peak at 19.7°. The relative crystallinity of the native starch is 38.8% (Guo et al. 2014; Salman et al. 2009; Tian et al. 1991). However, significant structural changes were observed in the triple modified starches. Specifically, the diffraction peaks at 15.3°, 18.3°, and 23.5° disappeared, while the peak at 17.2° was weakened. Additionally, a minor peak at 19.7° became more pronounced, and a new diffraction peak emerged at 13.1°. The disappearance of these three peaks, coupled with the retention of the 17.2° peak, suggests a distinct transition from a Ca‐type to a B‐type crystalline structure, indicating the disruption of the internal crystalline framework and gelatinization of starch granules (Guo et al. 2015). The peaks at 13.1° and 19.7° correspond to the formation of an amylose‐lipid complex, which is characteristic of a V‐type crystalline structure (Li et al. 2017). These results suggest that native sweet potato starch undergoes a transformation into a mixed crystalline form consisting of both B‐type (dominant peak at 2θ = 17.2°) and V‐type (major peaks at 2θ = 13.1° and 19.7°) structures, with the V‐type being the predominant crystalline form after enzyme modification. In this study, ethanol was used to precipitate the enzyme‐treated starches, leading to the formation of amylose or long‐chain starch‐ethanol complexes. Notably, all modified starch samples exhibited an intensified peak at 2θ = 19.7°, indicating the presence of an amylose‐ethanol complex (Biliaderis and Galloway 1989).

Compared to native starch, the modified starches exhibited lower relative crystallinity due to structural damage in the crystalline regions during enzymatic treatment. The relative crystallinity of swollen starch decreased from 38.8% to 14.8% as the degree of branching (DB) increased. At higher DB levels, a greater proportion of short linear glucans was present. These short‐chain glucans tend to form amylose‐ethanol complexes, which promote the development of a V‐type crystalline structure (Yan et al. 2021). The presence of B‐type crystallinity suggests looser molecular packing, which is often associated with higher digestibility and water absorption. The V‐type structure, meanwhile, indicates the formation of amylose‐lipid or amylose‐ethanol complexes, contributing to reduced retrogradation and better freeze–thaw stability. The decline in crystallinity can be attributed to the abundance of short‐chain glucans in the triple modified starches, as these short chains are unable to form stable double helices and, consequently, do not contribute to crystallinity (Witt et al. 2012).

These XRD results confirm that enzymatic modification transforms the crystalline structure of sweet potato starch from Ca‐type to a mixed B‐type and V‐type, contributing to altered functionality such as improved solubility and reduced retrogradation tendency.

### 3.4 Characterization of Hydrophilic Properties

**FIGURE 6:**
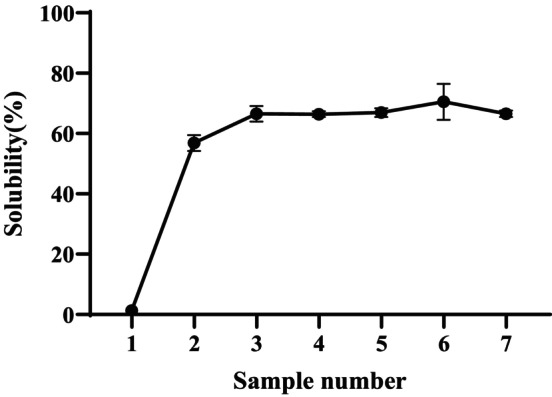
Solubility of the native and modified sweet potato starches with different DB (sample number 1–7) at 25°C. Sample 1: 16.99% (control), Sample 2: 27.93%, Sample 3: 32.30%, Sample 4: 38.00%, Sample 5: 41.87%, Sample 6: 45.91%, Sample 7: 53.38%.

Figure 6 presents the solubility of native and modified sweet potato starches measured at 25°C. Native sweet potato starch exhibits significantly lower solubility (1.32%) compared to the modified starch samples. Additionally, the solubility of the modified starches generally increases with rising DB values. This finding aligns with previous studies, which suggest that highly branched starches exhibit greater water solubility than normal starch, forming highly stable, clear solutions (Takata et al. 1996). The observed increase in solubility can be attributed to the enzymatic hydrolysis of native starch, which reduces its molecular size to a certain extent, thereby enhancing solubility (Jensen et al. 2013). More importantly, the introduction of branch points in amylose prevents recrystallization, further contributing to increased solubility. Additionally, the incorporation of branch points in amylopectin disrupts its highly organized structure, preventing glucan chain re‐alignment and further enhancing solubility Sievert and Wüsch (1993). It is well known that native starch is insoluble in cold water and has very limited solubility, restricting its practical applications. As shown in Figure 6, triple enzyme modification significantly improves starch solubility. Moreover, the solubility of modified starches increases with increasing DB, except for the sample with a DB of 53.38%. This anomaly may be attributed to an overly dense molecular configuration caused by excessive branching, which could hinder water penetration and limit hydration. This exception suggests that while solubility generally increases with DB, excessive branching may lead to a denser molecular structure, making highly branched starch molecules less soluble in water (Goesaert et al. 2010; Kowittaya and Lumdubwong 2014).

Therefore, starch solubility may be influenced by multiple factors, including the degree of branching, chain length, and branch linkage. However, the precise mechanism remains unclear and will be the focus of future studies.

### 3.5 Rheological Measurement

#### 3.5.1 Steady Shear Rheological Property

**FIGURE 7:**
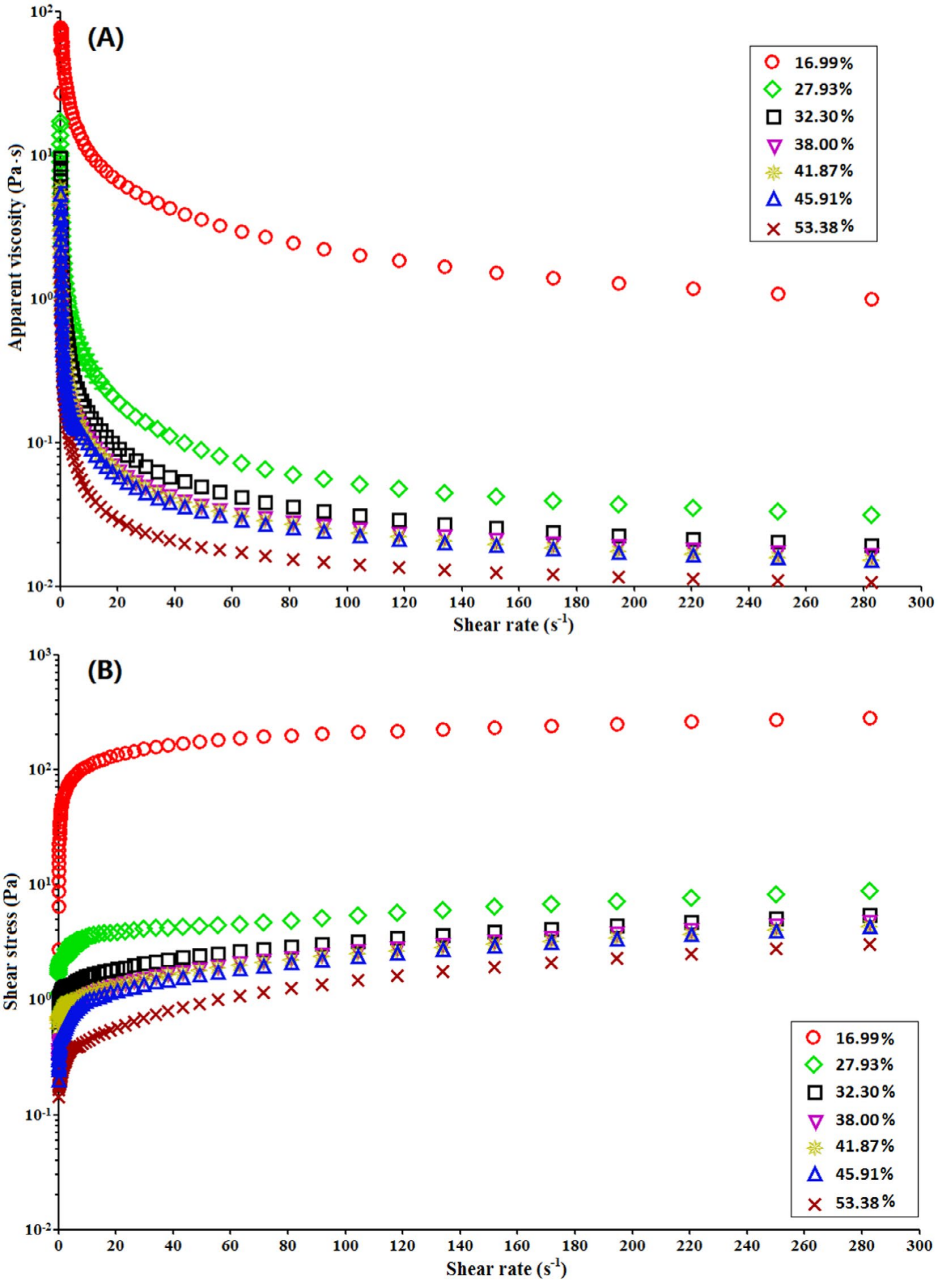
Steady shear properties of 6.0% native (DB = 16.99%) and modified sweet potato starch dispersions with different DB (27.93%–53.38%). (A) Apparent viscosity versus shear rate, (B) shear rate versus shear stress.

The flow behavior of native and modified sweet potato starch pastes at a concentration of 6.0% (w/v) at 25°C is shown in Figure 7. As illustrated in Figure 7A, the apparent viscosity of both native and modified sweet potato starch pastes decreases markedly with increasing shear rate, indicating that the starch suspension exhibits pseudoplastic (shear‐thinning) behavior. Compared to native sweet potato starch with a DB of 16.99%, the viscosity of modified starches with DB values ranging from 27.93% to 53.38% decreases significantly as DB increases. This phenomenon can be attributed to the fact that starch with a higher DB and shorter chain length experiences a greater reduction in viscosity (Ai and Jane 2015). Viscosity is a critical property of starch pastes, often limiting their practical applications due to excessively high viscosity. As depicted in Figure 7B, shear stress increases with shear rate, while the slope of the curve decreases as DB increases. Furthermore, both native and modified starch pastes exhibit a parabolic rheological profile, with the parabolic arc becoming less pronounced as DB increases. This suggests that both native and modified sweet potato starch pastes behave as non‐Newtonian fluids with pseudoplastic characteristics, although the pseudoplasticity is weakened at higher DB values.

#### 3.5.2 Dynamic Rheological Property

**FIGURE 8:**
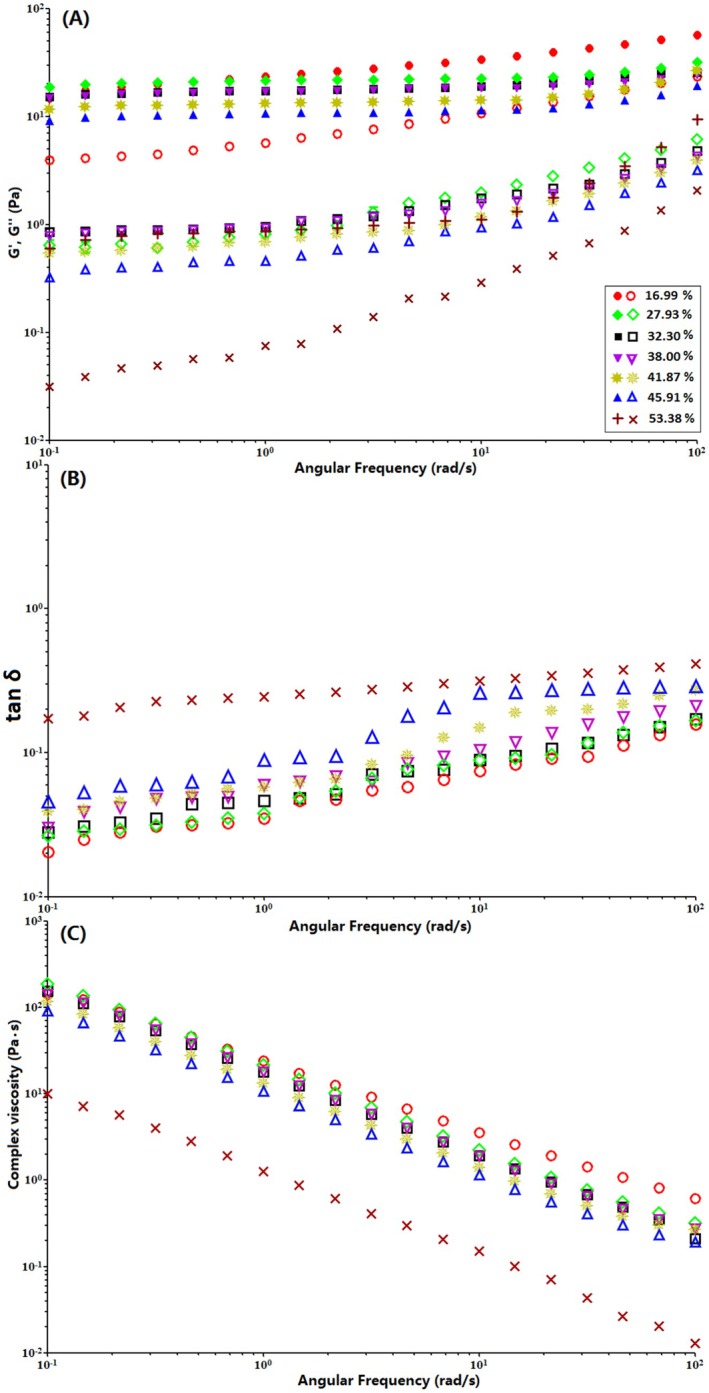
Variation of G′ and G″, tan δ, and η* of 6.0% native (DB = 16.99) and modified sweet potato starch dispersions with different DB (27.93–53.38) as a function of angular frequency.

Figure 8 illustrates the variations in storage modulus (G′), loss modulus (G″), complex viscosity (η*), and loss tangent (tan δ = G″/G′) as functions of angular frequency (ω) at 25°C for 6.0% (w/v) native and enzyme‐modified sweet potato starch pastes with different DB values.

As shown in Figure 8A, both G′ and G″ increase with increasing ω, indicating a frequency‐dependent behavior. In all samples, G' values are significantly higher than G″ values, suggesting the formation of a typical weak gel network with viscoelastic properties (Choi and Yoo 2008; Mohammed et al. 1998). The G′ and G″ values of modified starch pastes are considerably lower than those of native starch paste, and they decrease further with increasing DB, indicating that gel rigidity, strength, and viscoelasticity diminish as DB increases. Additionally, the frequency dependence of G′ and G″ is evident, with no crossover between the two moduli over the measured frequency range, further confirming the characteristics of a weak gel‐like three‐dimensional network (Ngwuluka et al. 2013). The loss tangent (tan δ = G″/G′) is commonly used to assess rheological changes resulting from ingredient interactions within a system. A tan δ value below 1 indicates a predominantly elastic material with solid‐like behavior, while a value above 1 signifies a more viscous material with liquid‐like characteristics (Takahashi and Fujita 2017). As illustrated in Figure 8B, the tan δ values for all starch samples remain below 1, confirming that their elastic nature dominates over their viscous properties. Furthermore, tan δ increases with increasing angular frequency, suggesting a shift toward more liquid‐like behavior, indicating that the three‐dimensional network becomes weaker and less gel‐like (Hsu et al. 2000). Enzyme‐modified sweet potato starch exhibits higher tan δ values than native starch, with tan δ increasing as DB increases. This trend suggests that enzyme modification leads to a higher proportion of viscous components and a reduction in elastic components compared to native starch. These results confirm that the enzymatic treatment inhibits amylose aggregation in sweet potato starch, consistent with previous findings (Li et al. 2017). Figure 8C presents the complex viscosity (η*) of native and modified starch pastes as a function of ω. The η* values of all samples exhibit a parallel decline in magnitude with increasing frequency, demonstrating the frequency‐dependent nature of η* in both native and modified sweet potato starches. Similar to the G′ and G″ trends in Figure 8A, modified starches display lower η* values than native starch, with η* decreasing as DB increases.

Overall, these results indicate that the elastic properties of both native and enzyme‐modified sweet potato starch pastes diminish at higher frequencies due to a reduced rate of molecular chain rearrangement (Gope et al. 2016).

## 4 Conclusion

This study successfully optimized the enzymatic modification of sweet potato starch to enhance its degree of branching (DB) using a combination of α‐amylase, β‐amylase, and transglucosidase. Response Surface Methodology (RSM) was employed to determine the optimal conditions, yielding a significant increase in DB while maintaining the granular structure of the starch. Structural analysis confirmed that enzymatic treatment resulted in a higher proportion of α −1,6 linkages, leading to improved solubility and reduced retrogradation tendencies. Rheological measurements indicated that modified starch exhibited lower viscosity and enhanced flow properties, making it more suitable for applications requiring reduced thickening properties. Additionally, XRD and FTIR analyses demonstrated alterations in crystallinity and molecular ordering, further supporting the successful modification of the starch structure. However, a potential limitation of this study is the uncertainty regarding the applicability of the optimized enzymatic conditions to other starch sources, such as maize, rice, or potato starch, which may differ in granular structure and enzyme sensitivity. Furthermore, the scalability of the sequential enzymatic process for industrial use requires further validation. Future work will focus on applying the modified starch in real food systems (e.g., sauces, dairy beverages) to assess its performance under processing conditions, as well as exploring alternative or synergistic enzyme combinations to further enhance branching efficiency and functional properties.

## Author Contributions

**Yang Jiao:** conceptualization (lead). **Pengtao Wang:** methodology (equal). **Lei Niu:** methodology (equal). **Rong Ai:** investigation (lead). **Liyun Xin:** data curation (equal). **Aili Song:** data curation (equal). **Guoxing Yang:** writing – original draft (equal). **Kai Zhang:** writing – review and editing (equal).

## Consent

The authors have nothing to report.

## Conflicts of Interest

G.Y. is affiliated with Gulang Tianyuan Agricultural Industry Co. Ltd., a company that produces flour. The other authors did not report conflicts of interest.

## Supporting information


**Table S1:** Summary of enzyme treatment conditions and corresponding DB values.

## Data Availability

The original contributions presented in the study are included in the article; further inquiries can be directed to the corresponding author.
