# Supplementary material for: Optimization of Enzymatic Parameters for Enhancing Branch Density and Flow Properties of Sweet Potato Starch
Source: Food Sci Nutr. 2025 Sep 1;13(9):e70822. doi: 10.1002/fsn3.70822 (PMC12402398; doi:10.1002/fsn3.70822)
Supplement: Supplementary file 1 — Table S1: Summary of enzyme treatment conditions and corresponding DB values. [file FSN3-13-e70822-s001.docx]

Table S1. Summary of enzyme treatment conditions and corresponding DB values.

|  | α(u/g) | β(u/g) | T(u/g) | t_α_(h) | t_β_(h) | t_T_(h) | DB(%) |
| --- | --- | --- | --- | --- | --- | --- | --- |
| 1 | 8.00 | 8.00 | 6000.00 | 2.00 | 6.00 | 12.00 | 21.00 |
| 2 | 8.00 | 8.00 | 6000.00 | 4.00 | 6.00 | 12.00 | 11.97 |
| 3 | 8.00 | 8.00 | 6000.00 | 6.00 | 6.00 | 12.00 | 15.07 |
| 4 | 8.00 | 8.00 | 6000.00 | 8.00 | 6.00 | 12.00 | 23.36 |
| 5 | 8.00 | 8.00 | 6000.00 | 10.00 | 6.00 | 12.00 | 25.92 |
| 6 | 8.00 | 8.00 | 6000.00 | 12.00 | 6.00 | 12.00 | 21.90 |
| 7 | 8.00 | 8.00 | 6000.00 | 10.00 | 2.00 | 12.00 | 19.21 |
| 8 | 8.00 | 8.00 | 6000.00 | 10.00 | 4.00 | 12.00 | 22.43 |
| 9 | 8.00 | 8.00 | 6000.00 | 10.00 | 6.00 | 12.00 | 29.39 |
| 10 | 8.00 | 8.00 | 6000.00 | 10.00 | 8.00 | 12.00 | 27.05 |
| 11 | 8.00 | 8.00 | 6000.00 | 10.00 | 10.00 | 12.00 | 25.60 |
| 12 | 8.00 | 8.00 | 6000.00 | 10.00 | 12.00 | 12.00 | 24.60 |
| 13 | 8.00 | 8.00 | 6000.00 | 10.00 | 6.00 | 2.00 | 24.43 |
| 14 | 8.00 | 8.00 | 6000.00 | 10.00 | 6.00 | 4.00 | 18.25 |
| 15 | 8.00 | 8.00 | 6000.00 | 10.00 | 6.00 | 6.00 | 24.60 |
| 16 | 8.00 | 8.00 | 6000.00 | 10.00 | 6.00 | 8.00 | 29.97 |
| 17 | 8.00 | 8.00 | 6000.00 | 10.00 | 6.00 | 10.00 | 32.37 |
| 18 | 8.00 | 8.00 | 6000.00 | 10.00 | 6.00 | 12.00 | 28.50 |
| 19 | 2.00 | 8.00 | 6000.00 | 10.00 | 6.00 | 10.00 | 14.09 |
| 20 | 4.00 | 8.00 | 6000.00 | 10.00 | 6.00 | 10.00 | 25.21 |
| 21 | 8.00 | 8.00 | 6000.00 | 10.00 | 6.00 | 10.00 | 29.53 |
| 22 | 16.00 | 8.00 | 6000.00 | 10.00 | 6.00 | 10.00 | 39.11 |
| 23 | 24.00 | 8.00 | 6000.00 | 10.00 | 6.00 | 10.00 | 30.04 |
| 24 | 32.00 | 8.00 | 6000.00 | 10.00 | 6.00 | 10.00 | 31.68 |
| 25 | 16.00 | 2.00 | 6000.00 | 10.00 | 6.00 | 10.00 | 46.52 |
| 26 | 16.00 | 4.00 | 6000.00 | 10.00 | 6.00 | 10.00 | 27.15 |
| 27 | 16.00 | 8.00 | 6000.00 | 10.00 | 6.00 | 10.00 | 28.23 |
| 28 | 16.00 | 16.00 | 6000.00 | 10.00 | 6.00 | 10.00 | 25.51 |
| 29 | 16.00 | 24.00 | 6000.00 | 10.00 | 6.00 | 10.00 | 27.09 |
| 30 | 16.00 | 32.00 | 6000.00 | 10.00 | 6.00 | 10.00 | 21.23 |
| 31 | 16.00 | 2.00 | 1500.00 | 10.00 | 6.00 | 10.00 | 54.09 |
| 32 | 16.00 | 2.00 | 3000.00 | 10.00 | 6.00 | 10.00 | 47.79 |
| 33 | 16.00 | 2.00 | 4500.00 | 10.00 | 6.00 | 10.00 | 36.69 |
| 34 | 16.00 | 2.00 | 6000.00 | 10.00 | 6.00 | 10.00 | 46.52 |
| 35 | 16.00 | 2.00 | 7500.00 | 10.00 | 6.00 | 10.00 | 46.14 |
| 36 | 16.00 | 2.00 | 9000.00 | 10.00 | 6.00 | 10.00 | 47.37 |
| 37 | 16.00 | 2.00 | 1500.00 | 10.00 | 6.00 | 10.00 | 37.60 |
| 38 | 12.00 | 2.00 | 2500.00 | 10.00 | 6.00 | 11.00 | 34.23 |
| 39 | 16.00 | 2.00 | 1500.00 | 10.00 | 6.00 | 10.00 | 35.74 |
| 40 | 20.00 | 3.00 | 1500.00 | 11.00 | 6.00 | 10.00 | 34.98 |
| 41 | 16.00 | 2.00 | 500.00 | 9.00 | 6.00 | 11.00 | 35.00 |
| 42 | 16.00 | 2.00 | 1500.00 | 10.00 | 6.00 | 10.00 | 36.06 |
| 43 | 16.00 | 2.00 | 2500.00 | 11.00 | 6.00 | 11.00 | 37.99 |
| 44 | 16.00 | 3.00 | 500.00 | 10.00 | 7.00 | 10.00 | 39.86 |
| 45 | 16.00 | 1.00 | 1500.00 | 10.00 | 5.00 | 11.00 | 41.74 |
| 46 | 16.00 | 2.00 | 500.00 | 11.00 | 6.00 | 9.00 | 38.72 |
| 47 | 16.00 | 1.00 | 500.00 | 10.00 | 5.00 | 10.00 | 40.94 |
| 48 | 20.00 | 1.00 | 1500.00 | 11.00 | 6.00 | 10.00 | 41.51 |
| 49 | 16.00 | 2.00 | 500.00 | 9.00 | 6.00 | 9.00 | 41.21 |
| 50 | 20.00 | 3.00 | 1500.00 | 9.00 | 6.00 | 10.00 | 42.85 |
| 51 | 20.00 | 2.00 | 500.00 | 10.00 | 6.00 | 9.00 | 39.92 |
| 52 | 16.00 | 3.00 | 1500.00 | 10.00 | 5.00 | 10.00 | 37.60 |
| 53 | 20.00 | 2.00 | 500.00 | 10.00 | 6.00 | 11.00 | 40.84 |
| 54 | 16.00 | 2.00 | 2500.00 | 11.00 | 6.00 | 9.00 | 34.03 |
| 55 | 12.00 | 2.00 | 500.00 | 10.00 | 6.00 | 11.00 | 37.87 |
| 56 | 16.00 | 3.00 | 1500.00 | 10.00 | 7.00 | 9.00 | 41.87 |
| 57 | 20.00 | 2.00 | 2500.00 | 10.00 | 6.00 | 11.00 | 37.69 |
| 58 | 16.00 | 3.00 | 2500.00 | 10.00 | 5.00 | 10.00 | 41.54 |
| 59 | 16.00 | 1.00 | 500.00 | 10.00 | 7.00 | 10.00 | 42.17 |
| 60 | 12.00 | 2.00 | 500.00 | 10.00 | 6.00 | 9.00 | 35.89 |
| 61 | 12.00 | 2.00 | 1500.00 | 9.00 | 5.00 | 10.00 | 35.79 |
| 62 | 12.00 | 3.00 | 1500.00 | 9.00 | 6.00 | 10.00 | 37.21 |
| 63 | 20.00 | 2.00 | 2500.00 | 10.00 | 6.00 | 9.00 | 38.98 |
| 64 | 12.00 | 2.00 | 1500.00 | 11.00 | 7.00 | 10.00 | 38.98 |
| 65 | 20.00 | 2.00 | 1500.00 | 9.00 | 7.00 | 10.00 | 32.30 |
| 66 | 16.00 | 1.00 | 1500.00 | 10.00 | 5.00 | 9.00 | 45.77 |
| 67 | 20.00 | 1.00 | 1500.00 | 9.00 | 6.00 | 10.00 | 43.19 |
| 68 | 16.00 | 3.00 | 1500.00 | 10.00 | 7.00 | 11.00 | 39.49 |
| 69 | 20.00 | 2.00 | 1500.00 | 9.00 | 5.00 | 10.00 | 43.08 |
| 70 | 16.00 | 2.00 | 1500.00 | 10.00 | 6.00 | 10.00 | 36.86 |
| 71 | 16.00 | 3.00 | 1500.00 | 10.00 | 5.00 | 9.00 | 45.91 |
| 72 | 16.00 | 1.00 | 1500.00 | 10.00 | 7.00 | 9.00 | 36.94 |
| 73 | 20.00 | 2.00 | 1500.00 | 11.00 | 5.00 | 10.00 | 33.97 |
| 74 | 16.00 | 3.00 | 500.00 | 10.00 | 5.00 | 10.00 | 36.46 |
| 75 | 16.00 | 3.00 | 2500.00 | 10.00 | 7.00 | 10.00 | 34.65 |
| 76 | 12.00 | 2.00 | 2500.00 | 10.00 | 6.00 | 9.00 | 34.88 |
| 77 | 12.00 | 3.00 | 1500.00 | 11.00 | 6.00 | 10.00 | 42.01 |
| 78 | 16.00 | 2.00 | 1500.00 | 10.00 | 6.00 | 10.00 | 38.00 |
| 79 | 12.00 | 1.00 | 1500.00 | 11.00 | 6.00 | 10.00 | 36.03 |
| 80 | 16.00 | 2.00 | 2500.00 | 9.00 | 6.00 | 9.00 | 40.76 |
| 81 | 12.00 | 2.00 | 1500.00 | 11.00 | 5.00 | 10.00 | 30.78 |
| 82 | 12.00 | 1.00 | 1500.00 | 9.00 | 6.00 | 10.00 | 33.40 |
| 83 | 20.00 | 2.00 | 1500.00 | 11.00 | 7.00 | 10.00 | 38.49 |
| 84 | 12.00 | 2.00 | 1500.00 | 9.00 | 7.00 | 10.00 | 27.93 |
| 85 | 16.00 | 1.00 | 1500.00 | 10.00 | 7.00 | 11.00 | 45.68 |
| 86 | 16.00 | 2.00 | 500.00 | 11.00 | 6.00 | 11.00 | 39.91 |
| 87 | 16.00 | 1.00 | 2500.00 | 10.00 | 7.00 | 10.00 | 35.65 |
| 88 | 16.00 | 2.00 | 2500.00 | 9.00 | 6.00 | 11.00 | 33.22 |
| 89 | 16.00 | 1.00 | 2500.00 | 10.00 | 5.00 | 10.00 | 43.00 |
| 90 | 16.00 | 2.00 | 1500.00 | 10.00 | 6.00 | 10.00 | 36.69 |
